# Supplementary material for: Exploring Cell Migration Mechanisms in Cancer: From Wound Healing Assays to Cellular Automata Models
Source: Cancers (Basel). 2023 Nov 3;15(21):5284. doi: 10.3390/cancers15215284 (PMC10647277; doi:10.3390/cancers15215284)
Supplement: Supplementary file 1 [file cancers-15-05284-s001.zip › cancers-2686740-supplementary.pdf]

Supplementary Materials

S1. Chemotaxis effect

In our work, only an isotropic condition is considered, but the chemoattractant effect can be involved by defining the chemoattractant-specific concentration gradient (SG) (Vasaturo et al., 2012) in the entire cell neighborhood. SG is calculated for each neighborhood site *i* as the normalized difference in concentration C with respect to the original site $\left(SG_i = \frac{C_i - C_0}{C_0}\right)$. The probability of migration to site *i* (P_i) depends on SG_i in the cell neighborhood, estimated according to an exponential average that uses a weighting parameter, γ, to describe cell line sensitivity to the concentration gradient (**3**) where N=9, including the eight neighborhood positions and the original position.

$$P_i = \frac{\exp\left(1-\gamma\cdot SG_i\right)}{\sum_1^N \exp\left(1-\gamma\cdot SG_i\right)} \qquad (3)$$

S2. Pearson correlation coefficient

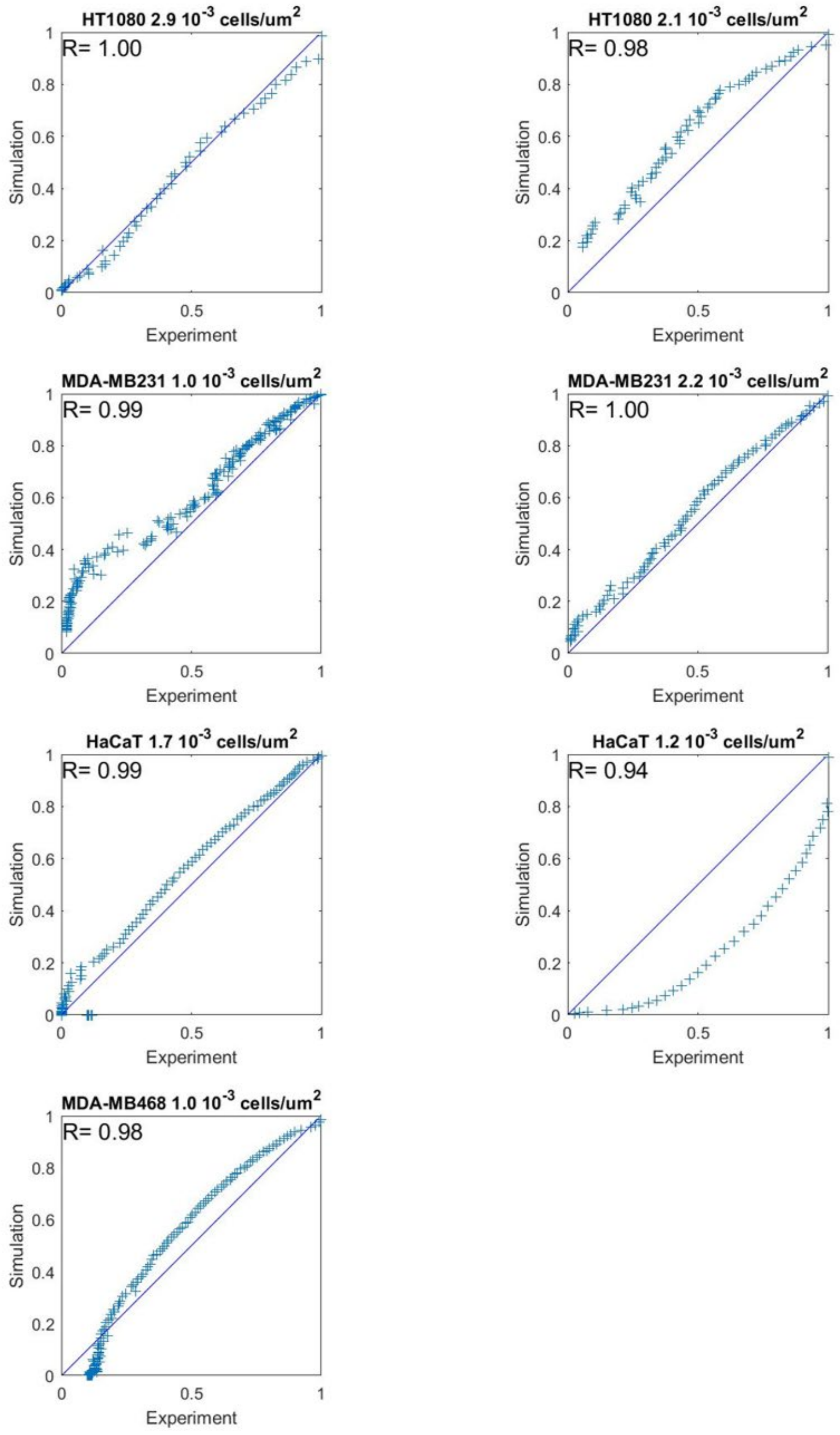

S2: Pearson correlation coefficients computed through *corrcoef* function in MATLAB. The input data are the ones provided in **Figure 3**.

S3. P-*value* histograms

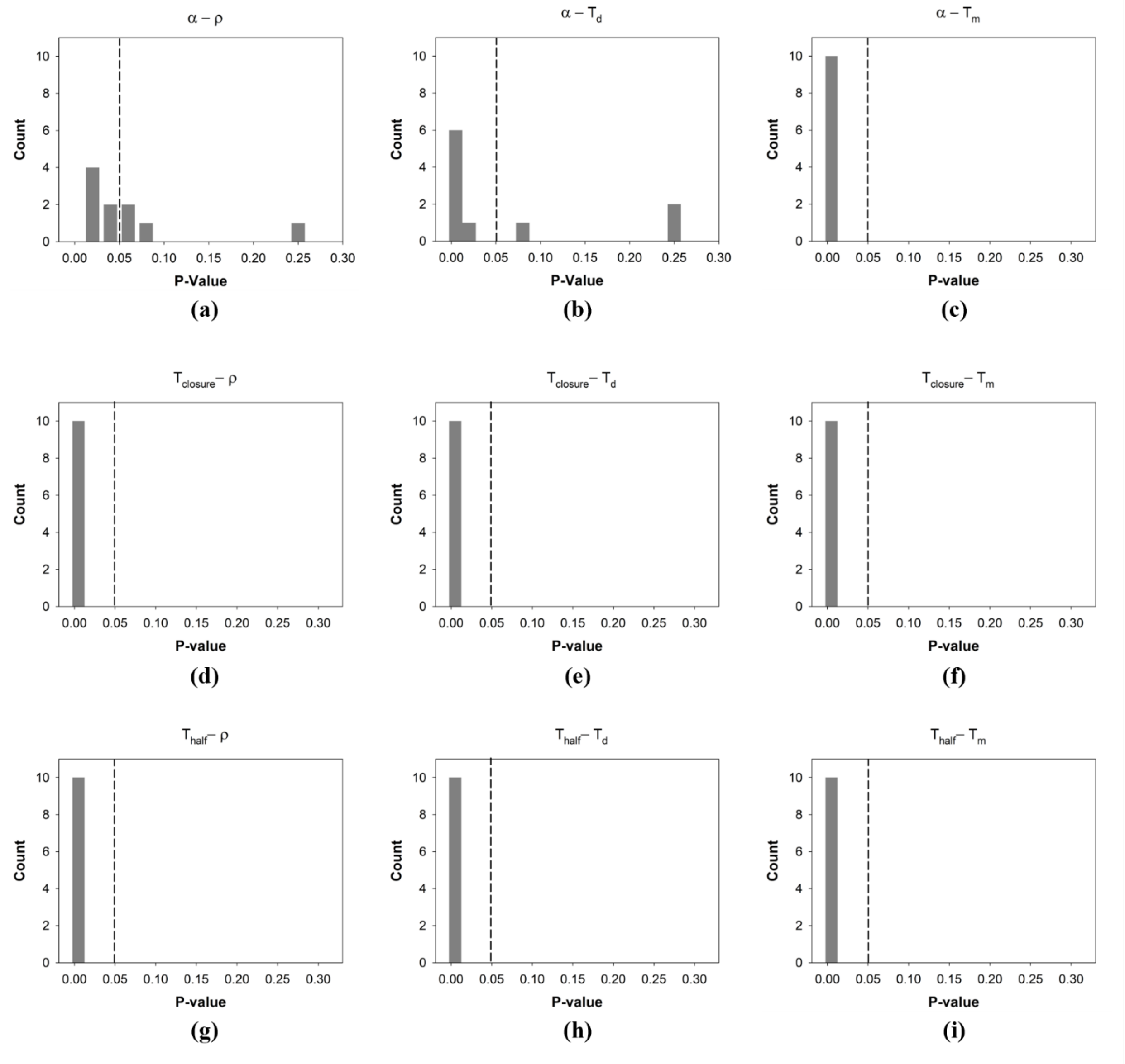

S3: Statistical analysis for MLRA. Histogram of p-value determined for 10 calculations of RC computed for α (a,b,c), $T_{closure}$ (d,e,f), and T_{half} (g,h,i). Data are considered significant for p-value<0.05.

S4. P-value histograms*: Physiologically valid ranges*

Looking at the range of the biological time of migration and proliferation (**Table 1**), according to the procedures in model development, the probability of proliferation has been computed for the extremes of the ranges.

1) T_m= 0.005 h; T_d= 12 h → P_d = 0.00012
2) T_m= 0.005 h; T_d= 40 h → P_d = 0.000125
3) T_m= 0.5 h; T_d= 12 h → P_d = 0.04
4) T_m= 0.5 h; T_d= 40 h → P_d = 0.0125

As the results show, in the hypothesis of a low-motility and high-proliferation cell line, the probability of proliferation in each time step is only 4%. This means that, just looking at the biological range, it can be concluded that migration is the governing parameter of the whole process.

S5. Convergence of number of simulations

To optimize computational costs (simulation times <5h) and admissible errors ($\varepsilon<5\%$), the cases of 700, 1000, 1700, and 2000 simulations were compared. In particular, the fitting parameters *a* and *b* in (**4**) were obtained by fitting five independent sets of simulations for each value # of the number of simulations. In the figure, the average value and the standard error of the mean are reported as error bars. By averaging five fits each from 2000 simulations, the error was limited below 5% (ε_a= 4.3% and ε_b= 0.1%).

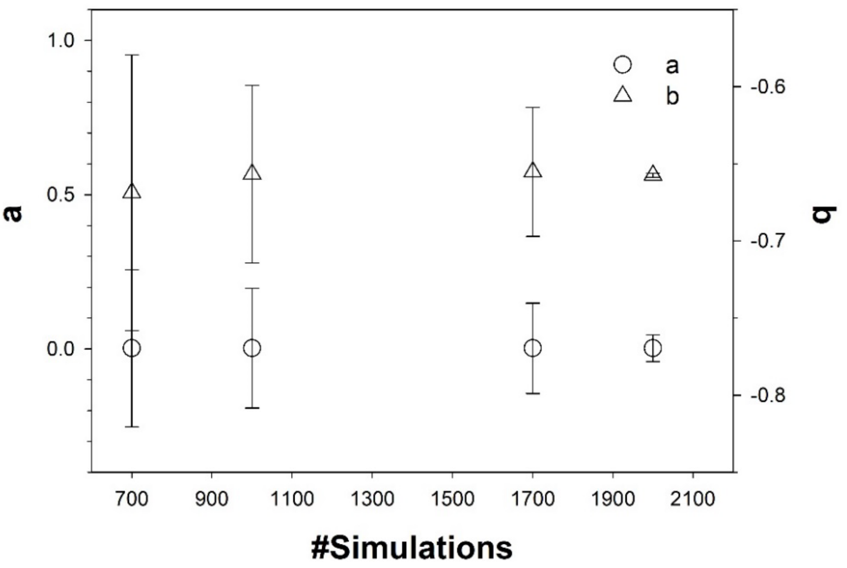

S5: Variation of fitting parameters with the number of simulations. Circles refer to parameter a (left axis), and triangles to parameter b (right axis). For each parameter reported, the error bar is obtained for three measurements.

S6. Estimate of input parameters from the literature.

Input parameters were estimated from the literature according to the following protocol:

- *Initial wound size b_o*

Given the values of α and v, b_0 is computed from equation $v = \alpha \cdot \frac{b_0}{2}$. In cases where the velocity of the front was not specified and the wound initial dimensions were unknown, b_0 was evaluated by image analysis of the figures reported in the paper.

- *Characteristic time of migration*

Knowing the typical doubling time of each cell line (T_d) from cell culture databases (e.g., ATCC, https://www.atcc.org/), the diffusivity constant is calculated using the invading-cell-front velocity predicted by Maini et al., 2004b, i.e., $D = \frac{v^2 T_d}{4\ln(2)}$. Since the characteristic time of migration is defined as the time needed by the cell to move a distance equal to its typical size (δ), it can be calculated as $T_m = \frac{\delta^2}{D}$.

- *Cell density*

The density values were estimated from figures reported in the papers with a careful analysis of images (using Image Pro Plus). The number of cells on the edges of the wound was counted and divided with respect to the area covered to estimate the cell density, ρ. Typically, a mean value is obtained by comparing the two edges of the wound.

S7. Phenomenological trend with fixed cell size δ

The value of fitting parameters (a and b in **Figure 4**) is estimated by varying all possible input parameters in the range reported in **Figure 5a**. In the case of a given cell line, variations in cell size δ can typically be considered to obtain better prediction accuracy. Additional simulations were realized with fixed cell sizes of δ = 20 and 10 mm. In the figure below, the two prediction curves are compared with experimental data from **Table 1**.

Comparing the two prediction curves, a crossing point can be identified for $\Phi = 0.1$, where a trend inversion is observed. For $\Phi < 0.1$, the closure velocity α in the case of smaller cells is lower with respect to the one predicted for bigger cells. This might be related to the fact that, in this range, motility is the limiting mechanism; thus, smaller cells take longer to cover the same space with respect to bigger ones. As the plot suggests, decreasing the ratio of characteristic times, i.e., very low values of Φ, will induce an increase in the velocity of wound closure; indeed, cells are characterized by high motility since T_m is low.

On the contrary, for $\Phi > 0.1$, our model predicts that the limiting process is proliferation, and, in this case, the closure rate of the wound for smaller cells is higher with respect to bigger ones. In addition, for higher values of Φ, α basically remains unchanged because the motility of cells is heavily reduced (high T_m). On the other hand, a reduction of T_d would reduce α but also have an impact on cell density and the increased cell–cell inhibition effect.

As discussed in the main text, the range of $\Phi > 0.1$ is at least rare to find in real physiological conditions; even assuming $T_d \cong 10$h, $\Phi > 0.1$ would require $T_m \cong 1$h. This is in agreement with our statement that, in physiological conditions, phenomena are always mainly driven by cell motility, even in the absence of proliferation inhibitors.

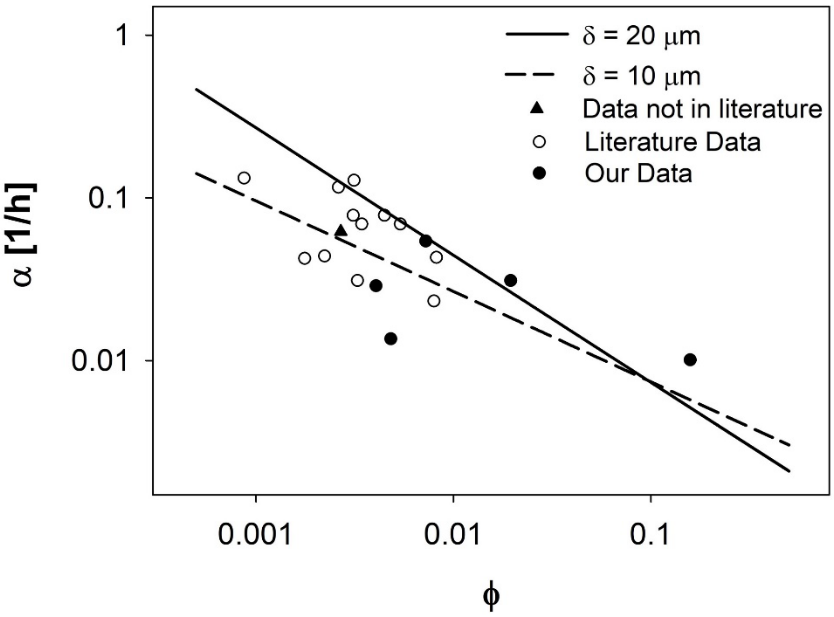

S7: The phenomenological trend is described by a straight line in the log scale, whose slope partially depends on cell size δ. Linear trends estimated by our analysis (continuous and dashed lines) are compared with experimental data (empty circles ○) evaluated from 12 different experiments (Ascione et al., 2017a; Ascione et al., 2017b; D'Argenio et al., 2012; Gaglione et al., 2017; Montano et al., 2019; and Vuoso et al., 2020 by our group and 6 further independent experiments obtained from the literature (Connolly et al., 2020; Jonkman et al., 2014; Lorie et al., 2020; Meir et al., 2015; Pautke et al., 2004; Tschon et al., 2015)) (filled circles •). The black triangle (▲) refers to additional data computed in our lab that is not in any publication. Details on the experimental parameters are reported in **Table 2.**
